# Abortion Ban Advocates and Rape Exception

**DOI:** 10.1007/s11673-024-10374-8

**Published:** 2024-09-24

**Authors:** Ł. Dominiak

**Affiliations:** Department of Social Philosophy, Faculty of Philosophy and Social Sciences, Nicolaus Copernicus University in Toruń, Fosa Staromiejska 1a, 87-100, Toruń, Poland

**Keywords:** Abortion, Prolife, Rape, Killing and letting die, Thomson, Boonin, Blackshaw

## Abstract

The present paper argues that abortion ban advocates can justify an exception for rape. Recently, Blackshaw offered an interesting argument that if abortion ban advocates modified their position along the lines of Thomson’s analysis of rights, they could make an exception for rape. However, doing so would require making concessions they would be unlikely to make, the crucial one being subscribing to an absurd view that abortion in the case of rape is permissible but only if it is performed in a certain way, that is, in a way that withdraws life support from the fetus. Agreeing first with Blackshaw’s premises, we argue that the view in question is hardly absurd. Thus, relying on Boonin’s acknowledgment that although very rare, a position according to which abortion should be legal but only if it is performed in a way that lets the fetus die rather than kills it is a possible position, we argue, first, that it is Blackshaw’s position that is inconsistent, second, that since deontology sees permissibility of a given result as path-dependent, deontologically oriented abortion ban advocates should find the view in question appealing rather than absurd and, third, that although there are indeed scenarios in which withdrawing life support is morally equivalent to killing, extraction abortions in the case of rape are not amongst them. Since in the case of rape the fetus is not entitled to life support, extraction abortions are better classified as permissible non-omissive allowings than impermissible killings. Thus, there is nothing absurd in the view that abortion in the case of rape is permissible but only if it is performed in a certain way. Accordingly, adopting this view does not seem to be much of a concession for abortion ban advocates who can therefore make an exception for rape.

## Introduction

In his recent paper, Bruce P. Blackshaw (2022) offered an interesting argument showing how abortion ban advocates could potentially make an exception for rape and thereby moderate their “extreme” position. As he argued, if they accepted some otherwise plausible assumptions about (1) rights, that is, Judith Jarvis Thomson’s (1971) account of what a right to life involves; and (2) responsibility, that is, Francis Beckwith (2006) as well as C’Zar Bernstein and Paul Manata’s (2019) arguments about the pregnant woman’s obligations stemming from consensual sex; and (3) showed that abortions can be regarded as withdrawing life support rather than killing, they could sustain the view that abortion in the case of rape is permissible. However, fulfilling the last condition would entail “what seems to be an absurd position for a prolife theorist—abortion in the case of rape is permissible, but *only* if the abortion is performed in a certain way” (Blackshaw 2022, 52). Since the alternative view (that could avoid this absurdity) that “in the case of the fetus, killing and letting die are equivalent” (Blackshaw 2022, 52) is also problematic as suffering from “further difficulties” (Blackshaw 2022, 52), then the most consistent position for abortion ban advocates is to maintain that abortion is impermissible also in cases of rape.

Now a curious thing about Blackshaw’s paper is that arguments offered in support of abortion ban advocates making an exception for rape are relatively compelling while reasons given for the ultimate conclusion that after all they should maintain that abortion is impermissible also in cases of rape seem much weaker. More specifically, Blackshaw convincingly argues that in the case of rape the fetus does not have a right to life support (although it has a right not to be killed) whereas in the case of consensual sex the pregnant woman has a duty to provide the fetus with such a support. Similarly, he makes a persuasive case against the view that killing and letting die are morally equivalent insofar as abortion is concerned. Put together, these arguments open up an interesting possibility for abortion ban advocates to attenuate their position by making an exception for rape: although in the case of non-rape pregnancies all abortions are impermissible, in the case of rape only abortions that kill the fetus are impermissible whereas abortions that consist in withdrawing life support are permissible. Nevertheless, Blackshaw rejects this promising possibility mainly because the view that “abortion in the case of rape is permissible, but *only* if the abortion is performed in a certain way” (Blackshaw 2022, 52) seems absurd. At the same time, he does not reveal any reasons for finding it absurd. In what follows we argue that there are actually good reasons for abortion ban advocates (including Blackshaw himself) to embrace this view and thus to make an exception for rape.

Our argument proceeds in the following way. Section "Blackshaw’s Argument" sets the stage for the later discussion by presenting both the gist of Blackshaw’s original argument and our point of departure in Boonin’s acknowledgement that although very rare, a position according to which abortion should be legal but only if it is performed in a way that lets the fetus die rather than kills it is a possible position. Section "Argument from Inconsistency" submits that Blackshaw’s position in its current form suffers from inconsistency. Avoiding this inconsistency constitutes the first good reason for Blackshaw and other abortion ban advocates to make an exception for rape. Blackshaw’s reluctance to do so seems to be mainly motivated by the belief that it would require subscribing to an absurd position that “abortion in the case of rape is permissible, but *only* if the abortion is performed in a certain way.” Section "Deontology and Path-Dependence" argues that this belief is unfounded since abortion ban advocates’ opposition to abortion is usually deontological in character and deontology sees permissibility of a given result as a function of the way in which it is brought about. Accordingly, there is nothing absurd about the view that permissibility of abortion is path-dependent. We posit that this fact constitutes the second reason for making an exception for rape. Section "Abortion and Withdrawing Life Support" considers more specific arguments put forward by Kate Greasley (2017, 47–51) and then employed by Blackshaw to the effect that even extraction abortions do not withdraw life support from the fetus but “involve an action being performed that results in the death of the fetus, and are still killing” (Blackshaw 2022, 51). These arguments undermine one of the steps that, according to Blackshaw, abortion ban advocates have to take in order to make an exception for rape, that is, the step consisting in showing that some abortions let the fetus die by way of withdrawing life support. As we argue, although extraction abortions indeed involve an action being performed and so cannot be regarded as pure omissions, it does not mean that they can therefore be regarded as killings. Much more pertinent subsumption is that they are double preventions or non-omissive allowings—the fact that constitutes, due to its moral import, the third reason for making an exception for rape. Section "Conclusions" concludes.

## Blackshaw’s Argument

In his recent paper on abortion, Blackshaw persuasively argues that anti-abortion theorists could make an exception for rape if they met three conditions: (1) accept Thomson’s argument that a right to life does not entail a right to life support, (2) demonstrate that Thomson’s analysis applies only to cases of rape but not to non-rape pregnancies and (3) show that abortions can be regarded as withdrawing life support. In order to justify the first condition, Blackshaw argues that abortion ban advocates, as pointed out by Matthew Scarfone (2021), cannot make an exception for rape on the basis of the argument that in the case of rape the pregnant woman’s right to bodily autonomy is invaded, for they also subscribe to the view that the fetus has a right to life and the latter right overrides the former, rendering abortions in the case of rape impermissible. Nevertheless, they can make their case for a rape exception in different terms than a conflict of rights. In this regard, Blackshaw quotes David Boonin’s (2002, 133–138) point that Thomson’s (1971) famous violinist argument is better construed as demonstrating that a right to life does not entail a right to life support than as contending that the pregnant woman’s right to bodily autonomy trumps the fetus’s right to life. Once it is established, a conflict between the fetus’s rights and the pregnant woman’s right to bodily autonomy disappears as the fetus simply lacks a right to use the pregnant woman’s body to start with. The fetus’s right to life, being a negative right as it is, correlates only with the pregnant woman’s negative duty not to kill the fetus, not with her positive duty to endure major sacrifices to support its life. Thus, unless the pregnant woman incurs such a duty in some other way, the fetus does not have a right to life support.

Although the fetus’s right to life does not entail the pregnant woman’s duty to support it, in cases of non-rape pregnancy, as pointed out by Blackshaw, the pregnant woman can incur such a duty on a different basis, that is, due to the fact that it is foreseeable for her that if she engages in sex, protected or not, she might get pregnant and thereby create a human being in need of her support. Thus, if she nonetheless decides to do so, she becomes responsible for the foreseeable consequences of her own behaviour, that is, for the fetus’s vulnerable existence. Drawing on this well-known responsibility objection to Thomson’s account, Blackshaw opposes Boonin’s arguments (the hedonist thought experiment) that the objection does not apply to pregnancies resulting from consensual sex and sides with such authors as Jeff McMahan (2002, 362–378), Beckwith (2006), and Bernstein and Manata (2019). Most crucially, Blackshaw invokes Bernstein and Manata’s (2019) cave diving thought experiment in which anytime a spelunker descends below some level in a cave, another person contracts a life-threatening kidney ailment that can be cured only by this person being hooked up to someone else’s circulatory system. Although spelunkers have helmets that warn them about descending too low, the equipment is not always reliable and some spelunkers decide not to use it anyway. Bernstein and Manata argue that spelunkers who are responsible for other people’s ailments have duties of assistance and so, once hooked up to these people, may not disconnect themselves. Now Blackshaw correctly points out that if spelunkers were forced to descend, they would not have such duties, for then their right to bodily autonomy, unattenuated by moral responsibility for other people’s ailments, would override any countervailing reasons to help these people. This argument allows Blackshaw to demonstrate that although the fetus’s right to life does not entail the pregnant woman’s duty to provide it with life support, the pregnant woman nonetheless incurs such a duty in cases of consensual sex. On the other hand, in cases of rape, she bears no duty to support the fetus.

Finally, even if in cases of rape the pregnant woman does not have a duty to provide the fetus with life support, she still has a duty not to kill it. Thus, if abortion kills the fetus, then the above reasoning is of no help to abortion ban advocates who would like to make an exception for rape. Needed is the third step, that is, showing that at least some abortions can be regarded as withdrawing life support. However, as pointed out by Blackshaw, there are two problems with such a view. First, it runs counter to Greasley’s (2017, 47–51) recent arguments that “in the vast majority of cases, abortion is a positive act of killing—it is not merely a withdrawal of life support or a failure to rescue” (Blackshaw 2022, 51) and even the so called extraction abortions “involve an action being performed that results in the death of the fetus, and are still killing, just as switching off a patient’s life support in an intensive care ward also is an act of killing” (Blackshaw 2022, 51). Second, even if abortion ban advocates could establish that extraction abortions only withdraw life support from the fetus rather than kill it, it would still entail “what seems to be an absurd position for a prolife theorist—abortion in the case of rape is permissible, but *only* if the abortion is performed in a certain way” (Blackshaw 2022, 52).

This absurdity could be avoided if it were showed that “in the case of the fetus, killing and letting die are equivalent” (Blackshaw 2022, 52). However, the view that killing the fetus is morally on a par with letting it die suffers from “further difficulties” (Blackshaw 2022, 52). Blackshaw identifies three such difficulties. First, he invites us to suppose, by analogy with a rape pregnancy, that a wicked man attached a heavy ball to our leg for nine months so that we cannot move freely. If we remove the ball an innocent person will die. Blackshaw correctly concludes that it is permissible to remove the ball, thereby letting the innocent person die, but impermissible to kill the innocent person. Embraced by Blackshaw, the solution to the wicked man scenario clearly speaks against the view that “in the case of the fetus, killing and letting die are equivalent” (Blackshaw 2022, 52). Second, Blackshaw, together with Daniel Rodger (Blackshaw and Rodger 2019), argues that “the killing versus letting die distinction explains why participating in induced abortions is far worse than failing to prevent spontaneous abortions” (Blackshaw 2022, 52–53). Adopting the equivalence view would block this explanation and commit abortion ban advocates to a problematic position that failing to prevent spontaneous abortions is almost as “seriously immoral” (Blackshaw 2022, 53) as participating in induced abortions. Finally, the same problem arises in the case of millions of children who die every year from preventable diseases and malnutrition. As Blackshaw points out, abortion ban advocates should think of themselves as morally responsible for these deaths “if there is no distinction between killing and letting die” (Blackshaw 2022, 53). For these reasons the view that “in the case of the fetus, killing and letting die are equivalent” (Blackshaw 2022, 52) is not really an option for abortion ban advocates.

Since both the position that “abortion in the case of rape is permissible, but *only* if the abortion is performed in a certain way” (Blackshaw 2022, 52) and the view that “in the case of the fetus, killing and letting die are equivalent” (Blackshaw 2022, 52) are problematic and come with further costs, Blackshaw concludes that the most consistent position for abortion ban advocates is to maintain that abortion is impermissible also in cases of rape. As he puts it, “both options seem unacceptable for a prolife view. The most consistent path for prolife theorists is to maintain that abortion is not permissible, irrespective of whether the pregnancy ensued from consensual sex or rape” (Blackshaw 2022, 53). Now a curious thing about this conclusion is that although Blackshaw gives us clear reasons for which the view that “in the case of the fetus, killing and letting die are equivalent” (Blackshaw 2022, 52) is too costly, he does not offer any reasons for which the view that “abortion in the case of rape is permissible, but *only* if the abortion is performed in a certain way” (Blackshaw 2022, 52) should be deemed absurd. Accordingly, it is hardly obvious that “the most consistent path for prolife theorists is to maintain that abortion is not permissible, irrespective of whether the pregnancy ensued from consensual sex or rape” (Blackshaw 2022, 53). To the contrary, if it were showed that the latter view is not absurd, then it would be exactly the position that abortion ban advocates who want to make a rape exception should assume as the most consistent one. After all, it is the view that logically follows from Blackshaw’s anti-abortion premises concerning the fetus’s right to life, the pregnant woman’s responsibility, and the non-equivalence of killing the fetus and letting it die.

Moreover, that such a view is indeed a possible perspective to assume can be gathered, for example, from Boonin’s (2019, 107–120) more recent discussion of the legality of abortion present in his book *Beyond Roe* where he considers a position according to which abortion should be legal but only if it is performed in a way that lets the fetus die rather than kills it. Although Boonin points out that it is a “pretty rare” (2019, 111) and “an unusual position to end up with, and one that won’t fully satisfy people on either side of the abortion debate” (2019, 112), he does not reject it but instead demonstrates that it would not contradict his own thesis that abortion should be legal—as this thesis “doesn’t have to mean [that] every method of abortion should be legal” (2019, 113)—and cautions its potential proponents that they would have to address a complication pertaining to the higher risks of the hysterotomy procedure (but not the abortion pill) which is one of the methods that fall under the rubric of letting the fetus die (2019, 116). Arguably, limiting this position exclusively to cases of rape pregnancies, as we propose to do in the present paper, might render it even rarer but perhaps also more acceptable for the abortion ban advocates as it supports only an exception to a general ban rather than a much broader permission. At any rate, relying on Boonin’s acknowledgment that it is indeed a possible position to take, we submit that it is also worth hearing. Thus, in the next two sections we argue that there are indeed good reasons for abortion ban advocates (including Blackshaw himself) to embrace this position and make an exception for rape.

## Argument from Inconsistency

Although Blackshaw (Blackshaw, Colgrove, and Rodger 2023, 128–130) is generally sceptical about arguments from inconsistency being deployed against abortion ban advocates, in a sense it is exactly the kind of argument that should be employed against Blackshaw in the current context. Thus, before we delve into substantive merits of the view that “abortion in the case of rape is permissible, but *only* if the abortion is performed in a certain way” (Blackshaw 2022, 52), let us first consider some formal aspects of Blackshaw’s reasoning.

As we saw above, Blackshaw’s argument proceeds from the premises that the fetus’s right to life protects the fetus only against killing and does not include a right to life support. The fetus acquires the latter right only if the pregnant woman engages in consensual sex, incurring thereby a correlative duty to support the fetus. However, in the case of rape, no such duty is created. Accordingly, in the case of rape, the fetus has a right not to be killed but no right to life support. From these two premises it follows as a matter of logic that in the case of rape killing the fetus is impermissible as it violates its right to life whereas withdrawing life support is permissible as it violates no rights of the fetus (neither the fetus’s negative right not to be killed nor its non-existent right to life support). Therefore, Blackshaw’s own premises entail that “abortion in the case of rape is permissible, but *only* if the abortion is performed in a certain way” (Blackshaw 2022, 52), that is, in a way that let the fetus die via withdrawing life support.

Now despite the fact that this is the view that follows from Blackshaw’s own premises, he refuses to accept it. In a sense, this is not an unfamiliar move in philosophy. If one tracks logical consequences of one’s reasoning and finds out that they lead to an absurd conclusion, one can avoid the absurdity by simply rejecting the conclusion. Thus, Blackshaw might be justified in disowning the view that “abortion in the case of rape is permissible, but *only* if the abortion is performed in a certain way” (Blackshaw 2022, 52). However, only if he also rejects at least one of his premises. After all, if his premises entail the conclusion that is absurd or false, then the falsity of the conclusion entails, by virtue of *modus tollens*, the falsity of the premises. One cannot have both: the truth of the premises and the falsity of the conclusion. The problem with Blackshaw’s reasoning is that he is unwilling to reject any of his premises.

Certainly, as a prolife theorist, Blackshaw is not ready to reject the premise that the fetus has a right to life. Neither is he prepared to circumvent this premise by adopting the view that “in the case of the fetus, killing and letting die are equivalent” (Blackshaw 2022, 52) in which case killing the fetus would not violate its right to life due to being morally on a par with letting it die. Similarly, Blackshaw firmly embraces the premise that in the case of rape it is permissible to withdraw life support from the fetus, for he subscribes to the view (Boonin’s interpretation of Thomson’s violinist argument) that the fetus’s right to life does not entail a right to life support and assumes the position that although the pregnant woman incurs a duty to support the fetus in the case of non-rape pregnancy (Bernstein and Manata’s spelunking thought experiment), she lacks any such duty in the case of rape pregnancy (Blackshaw’s own forced spelunking thought experiment). Thus, for Blackshaw, the truth of his premises seems to be beyond any doubt.

This fact calls into question consistency of Blackshaw’s argument. For as we pointed out above, as the truth of the premises travels forward to the conclusion, so the falsity of the conclusion travels backward to the premises. It is therefore difficult to make sense of a view which repudiates its own conclusion as absurd but refuses to reject at least one of its premises. Moreover, since Blackshaw’s reasons for embracing the premises seem relatively strong while his opposition to the conclusion is mainly based on the alleged absurdity thereof, then the identified inconsistency of Blackshaw’s argument speaks more against his rejection of the conclusion than against his premises. Accordingly, it seems that Blackshaw should subscribe to the view that “abortion in the case of rape is permissible, but *only* if the abortion is performed in a certain way” (Blackshaw 2022, 52) and make an exception for rape.

## Deontology and Path-Dependence

Besides the argument from inconsistency considered above, there are substantive reasons to believe that the view that “abortion in the case of rape is permissible, but *only* if the abortion is performed in a certain way” (Blackshaw 2022, 52) should not be deemed absurd by abortion ban advocates. On the most general level, these reasons are connected with the fact that the abortion ban advocates’ opposition to abortion is predominantly deontological in character. Certainly, abortion ban advocates do not oppugn abortion, at least not usually, on the grounds that it brings about less good than its prohibition. Rather, they formulate their criticism in terms of such moral absolutes as the sanctity of human life or inviolability of rights. It is therefore fair to say that abortion ban advocates’ opposition to abortion is usually based on deontological ethics (Żuradzki 2014).

Now deontological ethics sees permissibility of a given event as a function of the way in which it is brought about. As Leo Katz aptly puts it, for deontology, permissibility of a result is path-dependent: “the very same result, brought about by one path, is forbidden; brought about by another, however, it is acceptable” (Katz 1996, 56–57). Moreover, this path-dependence is commonly believed to be a necessary feature of deontological ethics.^1^ For example, writing about path-dependence, John L. Mackie points out that “the main reason why it has been thought important by Catholic moralists is that it seems necessary if there are to be absolute moral rules” (Mackie 1990, 161), for otherwise the deontological ethics which postulates such rules would collapse under “conflict cases where different rules, or even different applications of the same rule, require incompatible actions, so that the rule (or rules) cannot (both) be absolute” (Mackie 1990, 161).^2^

Consider in this regard the Trolley Problem made famous by Philippa Foot (1978, 23) and Thomson (1985). Whether it is permissible to kill one in order to save five depends on the way in which the one’s death is brought about. If you only redirect previously operating threat so that it kills the one instead of five, then killing the one is permissible. If you, on the other hand, initiate a new threat (by, for example, pushing the fat man onto the track or harvesting the check-up patient’s organs) that kills the one so that the five can escape a different threat, it is impermissible. Now there is nothing absurd about the view that killing the one is permissible but *only* if it is performed in a certain way. Quite the contrary, the Trolley Problem is the locus classicus of deontological ethics. Or take Thomson’s violinist thought experiment, even more pertinent in the current context. It is permissible for you to bring about the violinist’s death by disconnecting him from your body but impermissible to kill him by, for example, a bullet to the head. Again, it is hardly absurd to say that it is permissible to bring about the violinist’s death but *only* if it is brought about in a certain way. Similarly, according to the doctrine of passive euthanasia, it is permissible for the doctor, in certain circumstances, to switch off the respirator but impermissible to give his patient a lethal injection, not to mention shooting the patient. Or in ducking cases made famous by Boorse and Sorensen (1988), it is permissible for you to bring about your camping companion’s death by removing yourself from between him and a charging bear but impermissible to throw him into the bear’s claws. In all these and many other cases (Moore 2008) it is permissible to bring about another’s death *only* if it is brought about in a certain way.

Hence, on the most general level an argument can be made that since abortion ban advocates oppose abortion on deontological grounds and deontology treats the same results as either permissible or impermissible depending on the way in which they are brought about, then the view that “abortion in the case of rape is permissible, but *only* if the abortion is performed in a certain way” (Blackshaw 2022, 52), instead of being absurd, should be a natural position for abortion ban advocates to assume. Accordingly, abortion ban advocates should feel comfortable with making an exception for rape.

## Abortion and Withdrawing Life Support

On a more specific level, substantive reasons for accepting the view that “abortion in the case of rape is permissible, but *only* if the abortion is performed in a certain way” (Blackshaw 2022, 52) and so for making an exception for rape are connected with the fact that some abortions can indeed be regarded as letting the fetus die by way of withdrawing life support. To begin with, it is important to note that Blackshaw’s own opposition to the view in question does not seem to be based on the denial of the claim that some abortions can be regarded as letting the fetus die by way of withdrawing life support. Although he approvingly references Greasley’s (2017, 47–51) arguments that “in the vast majority of cases, abortion is a positive act of killing—it is not merely a withdrawal of life support or a failure to rescue” (Blackshaw 2022, 51) and that even extraction abortions “involve an action being performed that results in the death of the fetus, and are still killing, just as switching off a patient’s life support in an intensive care ward also is an act of killing” (Blackshaw 2022, 51), he does not seem to make much of these arguments. Instead Blackshaw suggests that even if abortion ban advocates proved Greasley wrong, it would still entail “what seems to be an absurd position for a prolife theorist—abortion in the case of rape is permissible, but only if the abortion is performed in a certain way” (Blackshaw 2022, 51). Thus, the gist of Blackshaw’s argument seems to be the alleged absurdity of the position in question, not the supposition that there are no abortions that only let the fetus die by way of withdrawing the life support.

Nevertheless, the substantive question of whether some abortions can indeed be regarded as merely letting the fetus die by way of withdrawing life support is worth considering in its own right as well as due to the fact that the negative answer thereto would make the entire discussion rather moot. Of course, taking into consideration the fact that the moral significance of the distinction between killing and letting die has been debated for a very long time and has already given rise to a substantial literature, it would be impossible for us to address it in a way that could satisfy all the participants in that debate. Accordingly, the following discussion will mainly speak to those who, like Blackshaw and deontologically oriented abortion ban advocates, tend to believe that there is some moral significance to the distinction between killing and letting die as applied to the question of abortion.

Thus, beginning with Greasley’s points approvingly referenced by Blackshaw (2022, 51) that, first, “many abortion procedures do not resemble the clean-cut ‘unplugging’ …, but involve something more like a direct attack on the body of the fetus” (Greasley 2017, 48), thereby constituting killing rather than letting die; and, second, that even extraction abortions which “do not involve a direct attack on the body of the fetus … are not *pure* omissions; in each case there is an action being performed which results in the death of the fetus” (Greasley 2017, 49) and so they are also better classified as killing than letting die, the same as some instances of terminating life-support “are clearly cases where disconnecting life-support without such an attack amounts to killing, not failing to rescue” (Greasley 2017, 49), two things should be said right away.

First, even if all abortions currently taking place were indeed killings, they would still be so only contingently, that is, depending on the existing incentives for health professionals to perform them in certain ways. For example, one could reasonably expect that if the moderate anti-abortion position became the law, abortion procedures would adjust instantaneously so as to avoid breaching it. Accordingly, in cases of rape pregnancies, abortions would cease to be positive acts of killing and become withdrawals of life support. Thus, the fact that currently “many abortion procedures do not resemble the clean-cut ‘unplugging’ …, but involve something more like a direct attack on the body of the fetus” (Greasley 2017, 48) and are therefore killings is inconsequential for the truth of the moderate anti-abortion position.

Second, although Greasley is clearly correct in saying that extraction abortions (such as abortion by hysterotomy, hysterectomy, or abortion pill), the same as switching off the life support in an intensive care unit, “are not *pure* omissions; in each case there is an action being performed” (Greasley 2017, 49), it would verge on a non sequitur to conclude from this fact that therefore the doctor causes the fetus to die, for an action involved might as well belong to a third category which Michael S. Moore (2009, 51–76) calls a double prevention (as it prevents the woman’s body from preventing the fetus’s death) or a non-omissive allowing (as it allows the fetus’s death by an act of removing the defence that the fetus has against the imminent death). In other words, instead of causally resulting “in the death of the fetus” (Greasley 2017, 49), it might simply result in removing the defence (the woman’s body) that the fetus has against the threat of death from its own inviability, just as switching off a patient’s life support in an intensive care unit involves an action that results in removing a defence (the life support system) that the patient has against the threat of death from an underlying condition. Or as ducking from a charging bear involves an action that results in removing a defence (your body) that your companion has against the threat of death from the charging bear. Neither of these cases involves an action that causes death—the death of the patient is caused by its underlying condition and the death of the camping companion is caused by the bear. By the same token, the death of the fetus is caused by its own inviability.

Now one could try to respond to this reasoning in one of two ways, that is, either by arguing that whereas switching off the life support or ducking a charging bear is indeed a double prevention, extraction abortions are instances of causing death or by accepting the analogy between switching off the life support and extraction abortions but arguing that since switching off the life support amounts to killing, so do extraction abortions. Beginning with the first response, one could argue^3^ that in the case of abortion there is a very intimate connection between the fetus and the woman’s body which renders it disanalogous with cases of ducking or switching off the life support. In the latter cases the connection between the protection and the party concerned is much more remote and thus it seems clearer that in removing the protection one does not *act on* or *use force on* the body of the party concerned. For example, my ducking a charging bear does not involve *acting on* or *using force on* the body of my camping companion. Even in the case of switching off the life support, the doctor does not *act on* or *use force on* the patient as he would if he were administering a lethal injection. Extraction abortions look different, for they primarily are acts of *expulsion*, that is, effectively shoving or forcing the fetus out of the woman’s body. Hence, it seems hard to deny that these abortions *act on* or *use force on* the fetus in order to remove it from the woman’s body. Arguably, there is no way to remove the protection supplied by the woman’s body without *acting on* the fetus itself and so it is much more difficult to regard extraction abortions as merely non-omissive allowings.

Even if we agree that extraction abortions do involve *acting on* or *using force on* the fetus, it does not follow that they are causings of the fetus’s death rather than double preventions, for what they are depends not so much on the question of whether they involve *acting on* or *using force on* the fetus as on the question of what this *acting on* or *using force on* the fetus causes—as *acting on* or *using force on* the fetus can either cause its disconnection from the woman’s body or its death. If it causes disconnection from the woman’s body due to which the fetus cannot sustain itself and dies of its own underlying inviability, then it is a double prevention and it does not matter that the disconnection was brought about by *acting on* or *using force on* the fetus rather than by, say, cutting the umbilical cord without even touching the fetus. The causal mechanism of the fetus’s death in both cases is the same. If, on the other hand, it causes, say, a collapse of the fetal skull rather than disconnection, then it is not a double prevention—but then it is not a typical extraction abortion either. Of course, when extraction abortions involve *acting on* or *using force on* the fetus, then besides causing the disconnection from the woman’s body, they also cause physical contact with the fetus and so can cause some injuries thereto as well. However, unless these injuries are mortal, they do not change the causal mechanism of the fetus’s death and the classification of extraction abortions as double preventions.

In this regard, compare again disconnecting the life support system in the intensive care unit. The first response to our analysis agrees, and in our opinion rightly so, that disconnecting the life support system is a double prevention. The typical causal mechanism involved here is that the doctor’s action causes the life support system which is preventing the patient’s death from the underlying condition to stop supplying its service by, say, switching off the ventilator or disconnecting the ventilator’s breathing tube—each action being sufficiently remote from the patient to say that the doctor does not *act on* or *use force on* the patient. Suppose, however, that the doctor causes the life support system to become disconnected from the patient by repositioning the patient or pushing him on another bed. It would be an outlandish or downright inappropriate way of disconnecting the life support that could even result in some legal consequences for the doctor, but it would be disconnecting the life support nonetheless. The causal mechanism of the patient’s death would be the same as in the typical cases. The doctor would cause the life support system which was preventing the patient’s death from another cause to stop supplying its service by disconnecting it from the patient although in a less remote way, that is, by *acting on* or *using force on* the patient. Of course, this *acting on* or *using force on* the patient, besides preventing the life support system from preventing the patient’s death from another cause, could at the same time cause some injuries (like bruises or abrasions) to the patient, but these minor injuries would not be causes of the patient’s death, the latter being whatever underlying condition the patient was suffering from. Accordingly, disconnecting the life support by *acting on* or *using force on* the patient would be as much a double prevention as disconnecting it by switching off the ventilator.

By the same token, the causal mechanism of the fetus’s death in at least some extraction abortions is that the doctor, by *acting on* or *using force on* the fetus, causes disconnection of the fetus from the woman’s body which was preventing it from dying of its own inviablity. If it were possible to do so entirely without *acting on* or *using force on* the fetus, the doctor would perform some act (say, cut the umbilical cord without even touching the fetus) that would cause the woman’s body to stop preventing the fetus’s death. But even when the doctor disconnects the woman’s body *by acting on* or *by using force on* the fetus, it does not change the fact that his *acting on* or *using force on* the fetus is only the cause of this disconnection, that is, of preventing the woman’s body from preventing the fetus’s death from another cause and not the cause of the fetus’s death (as, for example, a fetal intracardiac potassium chloride injection would be), the latter being the fetus’s own inviability.

This argument of course assumes that causing disconnection of the supporting system, be it the woman’s body or the ventilator, does not cause death of the party concerned but only prevents the supporting system from preventing death from another cause. It only establishes that *acting on* or *using force on* the party concerned does not in itself change this fact, for it can simply be a specific way of causing disconnection of the supporting system—as it is in typical extraction abortions—and thus still be an instance of double prevention. Yet, the assumption that causing disconnection of the supporting system is a double prevention rather than causing death can be questioned—which brings us to the second possible response to our argument.^4^

In recent years one of the most influential criticisms of this assumption in the context of withdrawing life-sustaining treatment was presented by Franklin Miller and Robert Truog (2012). Miller and Truog argued that withdrawing life-sustaining treatment from a patient causes his death because, following Foot (1994, 283), “it initiates the fatal sequence leading to death at the time it occurs” and “without the withdrawal, the patient would have continued to live” (Miller and Truog 2012, 4). This subsumption of withdrawing life-sustaining treatment under causing death rather than double prevention simply reflects what Miller and Truog, following Hart and Honoré (1985), call “our common-sense understanding of the causes of particular events” (Miller and Truog 2012, 6) according to which “causes are events or circumstances that *make the difference* in explaining a particular occurrence” (Miller and Truog 2012, 6). Thus, withdrawing life-sustaining treatment causes a patient’s death because it is an event, that is, an action rather than omission, that makes the difference to the occurrence of the patient’s death by initiating the fatal sequence of events without which the patient would have kept living.

However, as pointed out by Andrew McGee (2014, 34), it is not clear why we should regard the withdrawal of life-sustaining treatment as “the initiation of a *new* process, rather than as the *resumption* of a process that was stopped or suspended when the treatment was initially implemented,” especially taking into consideration the fact that, as Ben Bronner (2018, 54–55) reminded us, even “Foot explicitly denies this claim” and instead argues that in the case of withdrawing life support, “the fatal sequence resulting in death is not initiated but is rather allowed to take its course” (Foot 1994, 288). After all, the initial cause, say, a high-level spinal cord injury, for which the life-sustaining treatment had to be applied, did not cease to exist. Rather, only its effects, say, a respiratory failure and subsequent death, have temporarily been staved off. Thus, withdrawing the life-sustaining treatment does not cause or initiates a new spinal cord injury which leads to a respiratory failure which in turn leads to death. Instead, it simply allows the old cause, that is, the old spinal injury to develop its further biological effects, resuming thereby the old sequence of fatal events that was only temporarily suspended by the preventive measures. Or still differently, since what the application of the life-sustaining treatment to a patient with a high-level spinal cord injury does is to prevent his imminent death, then withdrawing the treatment merely terminates the prevention. As Bronner (2018, 49–50) puts it: “To provide aid to someone is to *prevent* them from suffering harm. To withdraw ongoing aid is to *stop* preventing them from suffering harm.”

Moreover, as pointed out by McGee (2014, 36-40), an argument can be made, following Helen Beynon (1982) and Lord Browne-Wilkinson’s opinion in Airedale NHS Trust v Bland (1993), that withdrawing life-support is causally inert vis-à-vis the patient’s death even on Miller and Truog’s own grounds. This problem is most visible in the case of artificial nutrition and hydration which is always provided periodically. If the doctors refrained from providing it first and only then withdrew the nasogastric tube, “the ‘withdrawal’ of the tube could *not* be a cause” (McGee 2014, 37) of the patient’s death. Certainly, withdrawing the tube could not make the difference to the occurrence of the patient death because he would die due to dehydration even if the tube were left in place. But neither could the doctor’s omission be such a cause, for Miller and Truog (2012, 13–14) reject, and rightly so, any causal powers of omissions (except when there is a duty to act—a problematic claim that we set aside here). Now “since refraining from providing the nutrition and hydration will normally precede the withdrawal of the tube, the so called ‘act’ of withdrawal will always be causally impotent” (McGee 2014, 37). Yet, as again pointed out by McGee (2014, 38), the same applies, *mutatis mutandis*, to withdrawing mechanical ventilation. For during changes of ventilators or airway tubes, the medical personnel ventilates the patient manually by squeezing a bag. Thus, if they refrain from squeezing the bag after a few squeezes, they will bring about the patient’s death by omission rather than action, rendering thereby the earlier act of disconnecting the ventilator “completely removed from the picture” (McGee 2014, 38), that is, causally inert vis-à-vis the patient’s death.

However, to our mind, the biggest problem with Miller and Truog’s argument is that it relies on what Bronner (2018, 46) called “causing as difference-making” and what we are prepared to call, again following Moore (2009, 287), causation as counterfactual dependence. Now in our present analysis, as well as in general, we reject the counterfactual reading of causation as fundamentally flawed. Although this is not the time and place for a detailed discussion of this complex matter, it is worth mentioning that even Hart and Honoré on whom Miller and Truog (2012, 6) rely in their understanding of causation believed that “[p]lainly, to be a condition *sine qua non* of some event on some given occasion and to be causally connected with it are not the same thing” (Hart and Honoré 1985, 121–122). Moreover, Moore (2009, 391–425) gives us no less than nine fundamental reasons for rejecting the counterfactual reading of causation, including such problems as the existence of non-causal counterfactuals, the notorious overbreadth of counterfactual dependence (for example, the counterfactual dependence between epiphenomenal events or omissions as counterfactual causes) or sundry types of overdetermination (symmetrical, asymmetrical, mixed-concurrent, or pre-emptive overdetermination). Some of these reasons play out quite straightforwardly in the problems besetting Miller and Truog’s arguments. For example, Miller and Truog try to alleviate the overbreadth problem by postulating the act requirement in order to discount the causal powers of omissions (admission of which would undermine their argument) even though it is quite clear that the doctor’s omission to provide the life-sustaining treatment does make the difference to the occurrence of the patient death. Similarly, the overbreadth problem is also visible in cases such as Bronner’s (2018, 48) ingenious do-not-resuscitate order scenario in which a doctor appropriately writes down a do-not-resuscitate note and the patient dies. As pointed out by Bronner (2018, 48), “writing of the order is an *act* that *makes the difference* to whether the patient lives or dies. Hence, the doctor causes the patient’s death if causing is understood as difference-making. Yet, there is no question whether the doctor has violated the prohibition on causing the death of patients.” Thus, Miller and Truog’s reading of causation as difference-making predicts that there is causation where there is none, clearly suffering from the overbreadth problem. All in all, one can say that as Miller and Truog criticize other authors, and rightly so, for cherishing a moral bias in their causal analyses by conflating moral responsibility and causation while “it is clear that these concepts are logically distinct” (Miller and Truog 2012, 12), they should themselves be criticized for cherishing a different, counterfactual bias in their own analysis, for it is equally clear that difference-making and causation are distinct phenomena. Thus, we conclude—together with McGee (2014, 46–47) and Bronner (2018, 61)—that Miller and Truog did not succeed in showing that withdrawing life-sustaining treatment amounts to causing death. Accordingly, if the analogy between switching off the life support and extraction abortions is accepted—and as we argued above, it should—then extraction abortions do not amount to causing death either.

Now Greasley’s position can also be seen as a variant of Miller and Troug’s criticism of what Bronner (2018, 44) calls the Standard View, for it also questions the assumption that causing the disconnection of the life support system, be it the woman’s body or the ventilator, is a double prevention rather than killing. However, instead of focusing predominantly on the issue of whether such a disconnection causes death, Greasely draws more heavily on the question of its moral import. Thus, Greasley agrees that “extraction abortions do not involve a direct attack on the body of the fetus” (Greasley 2017, 49). Nevertheless, she argues that this fact alone does not settle the question of whether extraction abortions only allow the fetus to die, “for there are clearly cases where disconnecting life-support without such an attack amounts to killing, not failing to rescue” (Greasley 2017, 49). The scenario that she offers in support of her claim is the case in which “a hospital attendant decides to switch off all of the life-support machines on an intensive-care ward without relocating the patients depending on them” (Greasley 2017, 49). This, as Greasley points out, would “surely be killing the patients and not just omitting to save them. As in this example, the abortion procedures in question are not *pure* omissions; in each case there is an action being performed which results in the death of the fetus” (Greasley 2017, 49).

However, there are two problems with this analysis. First, if to kill is simply to cause the death of another—as we, again following Moore (2009), believe it is—then neither extraction abortions nor switching off the life-support machines kill the parties in question, for as we argued above, neither cause their death. Rather, what these actions effectuate is removing defenses that these parties have against the imminent death from, respectively, inviability and diseases. This, on the other hand, is not to say that these double preventions are not, at least sometimes, *morally* equivalent to causing death or killing, if you will—and this realization brings us to the second and crucial problem with Greasley’s analysis.

Thus, although it is true that the hospital attendant commits an act that is morally equivalent to killing, the fact that he performs an action that prevents the life-support machines from preventing the death of the patients is hardly the only factor that drives this conclusion. Another driving factor is that the patients are entitled to life support and that he—as a hospital attendant—has a special duty to provide them with such a support. Suppose they were not so entitled. For example, suppose that the hospital attendant switched off a life-support machine that he was providing to a poor life quality patient in order to save a higher life quality patient or two such patients. Then we would be unwilling to conclude that he surely killed the former patient. Compare it now with a case in which the hospital attendant gives a poor life quality patient a lethal injection in order to distribute the life-support machine to a higher life quality patient. Now our willingness—as well as law’s willingness—to conclude that he surely killed the former patient would be much stronger. Therefore, what drives the moral conclusion in Greasley’s original hospital attendant case is both the fact that the hospital attendant performed an action that prevented the life-support machines from preventing the death of the patients and the fact that the patients were entitled to life support. Eliminate the second condition and the moral conclusion changes drastically. Replace the first condition with causing death and even if the second condition is not met, the moral conclusion changes as well (Moore 2009, 59-65).

To sum up, we agree with Greasley that although “extraction abortions do not involve a direct attack on the body of the fetus, … there are clearly cases where disconnecting life-support without such an attack amounts to killing” (Greasley 2017, 49). However, not being an instance of causing death of another (and thus not amounting ontologically to killing), disconnecting life support amounts morally to killing only if there is an entitlement to life support to start with. Since in the case of rape pregnancies—as Blackshaw argued in his paper—the fetus does not have a right to life support, extraction abortions which only remove the defence that the fetus has against the threat of death from its own inviability, do not amount morally (nor ontologically) to killing the fetus. It therefore seems that “abortion in the case of rape is permissible, but *only* if the abortion is performed in a certain way” (Blackshaw 2022, 52), that is, in a way that removes life support from the fetus rather than causes its death.

## Conclusions

We argued that the view, entailed by Blackshaw’s own premises, that abortion in the case of rape is permissible but only if performed in a way that withdraws life support from the fetus is hardly absurd and abortion ban advocates might actually have good reasons to adopt it. However, the above discussion certainly leaves out many issues—including, for example, responsibility of the rapist for the death of the fetus, the fetus’s right to life failing to trump the woman’s right to bodily autonomy when the latter is invaded by rape or a more comprehensive treatment of the distinction between killing and letting die and its application specifically to the question of abortion in cases of rape pregnancies—that would have to be addressed in order for us to offer a more thorough defence of the position deemed absurd by Blackshaw. Due to the space limitations, we were unable to address them in an adequate way in this place. Accordingly, we have to stay content, even if only partially, with the above justification based on Boonin’s acknowledgment that a version of the view defended in this paper is a rare but possible position to have, inconsistency of Blackshaw’s own argument, the path-dependent nature of the deontological opposition to abortion, some arguments for classifying extraction abortions as non-omissive allowings rather than causings and the assumption that such a classification matters morally.

## Data Availability

I do not analyse or generate any datasets, because my work proceeds within a theoretical approach. One can obtain the relevant materials from the references below.

## Footnotes

1 For example, Michael S. Moore (1997, 453), while himself embracing path-dependence of deontological prohibitions, mentions Elizabeth Anscombe, John L. Mackie, Charles Fried, and Heidi M. Hurd as proponents of this thesis.

2 According to Mackie, one way in which deontological prohibitions are path-dependent is that they “distinguish positive acts from omissions, and frame absolute rules only about positive acts. No conflict cases could then arise, because in any conceivable set of circumstances all prohibitions of kinds of positive act … could be obeyed at once by complete inaction” (Mackie 1990, 162).

3 This argument was put forth as a challenge to our view by an anonymous referee of this journal and so we present it almost verbatim in order to not misrepresent it. We are grateful to the referee for pushing us on this point as responding to it renders our argument clearer and more comprehensive.

4 This response was suggested by another anonymous referee of this journal. We are grateful to the referee for inducing us to delve deeper into the problem of withdrawing life-sustaining treatment as it stands vis-à-vis the killing/letting die distinction.
